# The Molecular Mechanism of Rhein in Diabetic Nephropathy

**DOI:** 10.1155/2014/487097

**Published:** 2014-11-11

**Authors:** Cong-Cong Zeng, Xi Liu, Guo-Rong Chen, Qian-Jia Wu, Wang-Wang Liu, Hai-Ying Luo, Jin-Guo Cheng

**Affiliations:** ^1^Department of Nephrology, The Wenzhou Traditional Chinese Medicine Affiliated Hospital of Zhejiang Chinese Medical University, Wenzhou, Zhejiang 325000, China; ^2^Department of Pathology, The First Affiliated Hospital of Wenzhou Medical University, Wenzhou, Zhejiang 325015, China

## Abstract

Diabetic nephropathy (DN) is characterized by unclear pathogenesis. Recent medical data shows that the incidence of DN rises year by year. Rhein is the main compositions of rhubarb, a traditional Chinese medicinal plant, which plays an active role in kidney protection. The prophylaxis and phytotherapeutic effects of rhein are due to its anti-inflammatory and antifibrosis properties. Here, we shed light on the renal protective role of rhein in diabetes mellitus (DM) with a particular focus on the molecular basis of this effect.

## 1. Introduction

Diabetes mellitus (DM) shares a significant medical burden all over the world with its high incidence and numerous complications [1]. Poorly controlled blood glucose leads to the occurrence and development of complications in patients with DM [2]. As one of the most serious diabetic microvascular diseases, diabetic nephropathy (DN) is the leading cause of end-stage renal failure [3, 4]. However, we have not found the exact pathogenesis of diabetic nephropathy, bringing certain difficulty to cure. Therefore, to explore the exact and feasible drug is current research hotspot and trouble in medicine.

**Figure 1:**
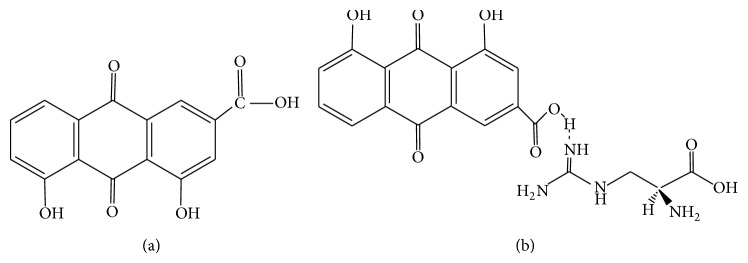
The chemical structure of rhein (a) and argirein (b).

In 1980s, rhubarb, a traditional Chinese medicinal herb, was first used to treat the patients who acquired DN by a Japanese scholar [5]. Hence, ample studies find the major renoprotective components of rhubarb are rhein, emodin, and chrysophanol, especially rhein [6, 7]. Rhein (4,5-dihydroxyl-2-carboxylic-9,10-dihydrodiketoanthracene.  Figure 1(a)) a free anthraquinone compound isolated from rhubarb, has extensive pharmacological actions, including the antibacterial, antiviral, anti-inflammatory, antiproliferative, and antifibrosis properties [8]. Although rhein has been widely used to treat numerous diseases in model animals [9–12] during recent decades and many studies have also revealed its pharmacological functions and mechanisms, no comprehensive article has yet been showed on DN. In this review, we summarize* in vivo* and* in vitro* experiments that suggest that rhein mediates multiple molecular targets implicated in DN.

## 2. The Pathogenesis of Diabetic Nephropathy

The pathophysiological performances of DN include renal hypertrophy, glomerular and tubular basement membranes thickening, mesangial matrix expansion, ultimately renal glomerular fibrosis, and sclerosis [13]. There are a lot of pathogenic mechanisms that (Table 1) can contribute to the development of this disease, such as glucose and lipid metabolism disorders, hemodynamic dysfunctions, abnormally expressed cytokines, oxidatives stress mechanisms, and genetic susceptibility [14].

## 3. Hypoglycemic and Hypolipidemic Benefits of Rhein

Rhein turned out to attenuate the glucose and lipid metabolism disorders. For example, in diet-induced obese mice, rhein shows good antihyperglycemic effect and lipid-lowering activity [15]. Besides, Zheng et al. use MCGT1 cells, a glucose transporter 1 (GLUT1) transgenic rat mesangial cell line, as a model to mimic mesangial cells in diabetic conditions, to elucidate that rhein can bring down the glucose levels in MCGT1 cells and the expression of GLUT1 by inhibiting the increased activity of the hexosamine pathway [16]. The increased activity of the hexosamine pathway has been considered as a key element involved in the metabolic disturbances of diabetes [17]. The expression of GLUT1 is obviously increased in patients with DN, and its expression intensity correlates with kidney disease severity. Not only so, the excessive expression of GLUT1 may activate hexoseamine pathway, followed by the production of TGF-*β*1. In accordance with the above theory, another study found that rhein markedly downregulates TGF-*β*1, thereby reducing GLUT1 mRNA expression in mesangial cells [18]. Rhein was also found to inhibit peroxisome proliferator-activated receptor gamma (PPAR*γ*) signaling [19] and suppress the expression of sterol regulatory element-binding protein-1c (SREBP-1c) [20], leading to blocking high-fat diet-induced obesity and decreasing fat mass and lower serum cholesterol and LDL cholesterol in the mice, and ameliorating lipid metabolism. In addition, a near research revealed that the mitochondria fission/fusion modulator-dynamin-related protein 1 (Drp1) plays an important role in promoting hyperglycemia-induced apoptosis of *β*-cells, while rhein reversed the expression of Drp1 and then largely localized at mitochondria in the *β*-cells and strongly protected pancreatic *β*-cells from hyperglycemia-induced apoptosis [21]. Moreover, rhein has been proved to improve insulin secretory function of pancreatic *β*-cells by preservation of *β*-cell mass and inhibition of *β*-cell apoptosis and enhance the ability of glucose tolerance [22].

## 4. Anti-Inflammatory Benefits of Rhein

Recently, a large body of studies elucidate that inflammatory responses are vital to the pathogenesis of diabetic nephropathy. Rhein, a natural medicine extracted from rhubarb, is proved to have an anti-inflammatory effect in animals and the clinic [23, 24]. Rhein is characterized by downregulating proinflammatory cytokines and signal transducers, such as VCAM-1, activator protein (AP-1), NF-*κ*B, MMPs, and MEK/ERK dependent pathways. Linlin Peng's results indicate that rhein could effectively suppress integrin-linked kinase (ILK) expression and regulate abnormal matrix metalloproteinase-9/tissue inhibitor of metalloproteinase-1 ratio (MMP-9/TIMP-1) in HK-2 cells and inhibit the progress of epithelial-mesenchymal transition (EMT). These inhibitory effects of rhein are due to the ILK suppression [25].

Also, it is demonstrated that rhein effectively suppresses the overexpression of fibronectin and TGF-*β*, thus reducing accumulation of extracellular matrix, protecting renal function, and ameliorating renal histological changes [26, 27]. Furthermore, argirein (Figure 1(b)), a supermolecule derived from chemical modification of rhein by combining with L-arginine through hydrogen bond (Figure 1(b)), is effective in suppressing the proinflammatory cytokines contributing to the pathogenesis of DN dependent on both anti-inflammatory activity of rhein and the NO offering activity of L-arginine. Argirein attenuates diabetic nephropathy in streptozotocin-injected rats through suppressing upregulated communication molecule Cx43 (connexin 43) but improving the depressed expression of PPAR*α* remarkably in renal tissue [28].

## 5. Antioxidant Benefits of Rhein

Although the precise mechanism of DN is still ambiguous, oxidative stress has been deemed as a central mediator in promoting the progression of nephropathy in patients who have diabetes. Rhein's unique structure that contains some powerful polar groups, including one carboxyl and two hydroxyls, contributes to its benefits of antioxidant [29]. Excessive production and generation of reactive oxygen species (ROS) induced by sustained hyperglycemia are a crucial contributor underlying the pathogenesis of diabetes associated with macrovascular and microvascular complications including diabetic nephropathy. While Heo et al.'s finding [30] that rhein could decrease ROS production and NADPH oxidase p47(phox) activation proves the antioxidant properties of rhein, at the same time, this discovery provides the basis for rhein becoming an antioxidant possessing promising therapeutic potential in disrupting the development of DN. Also, in an experiment which uses hydrogen peroxide (H_2_O_2_) to induce injury in human umbilical vein endothelial cells (HUVECs), rhein significantly increased the viability of H_2_O_2_-injured HUVECs by decreasing the malondialdehyde (MDA) and lactate dehydrogenase (LDH) content, increasing the nitric oxide (NO) content and nitrogen oxide synthase (NOS), superoxide dismutase (SOD), and glutathione peroxidase (GSH-PX) activity [31]. In the according article occurring before [28], it simultaneously illuminated that the mRNA and protein distribution of NADPH oxidase (subunits of p22phox, p47phox, and p67phox) in renal tissue may be mitigated by antioxidant activity of argirein.

**Figure 2:**
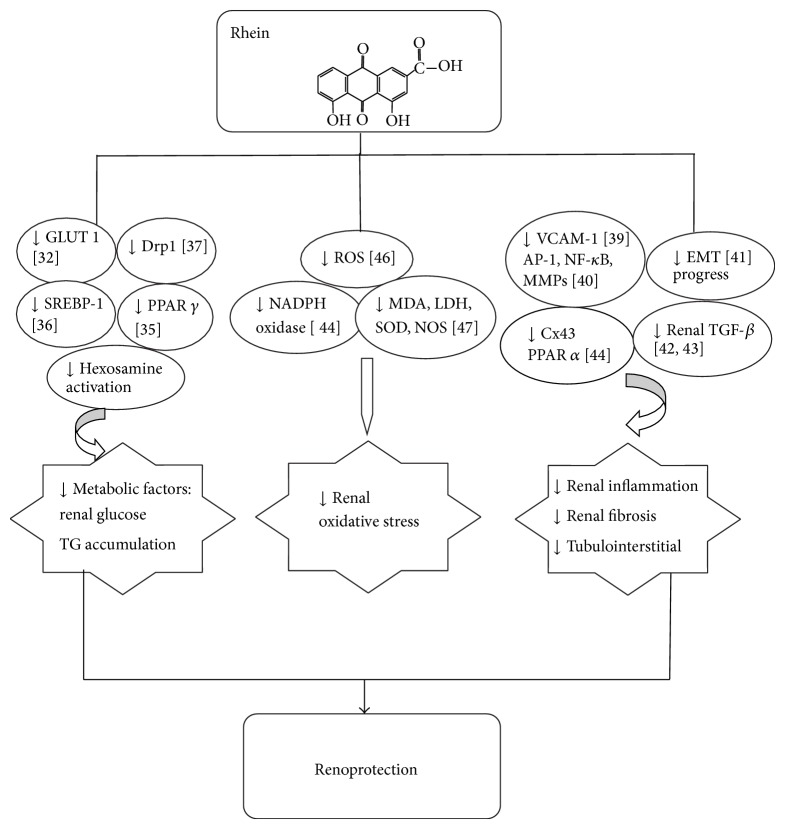
Possible mechanisms of rhein works on the renal protection.

To sum up, rhein can suppress hyperglycemia, hyperlipidemia, inflammation, and oxidative stress, interacting with multiple molecular targets, therefore improving the pathological performance happening in the progress of diabetic nephropathy (Figure 2).

## 6. Pharmacokinetic Studies and Clinical Trials of Rhein

Because of its beneficial influence on diabetic nephropathy, rhein may be an efficacious antidiabetic nephropathic drug. In order to have more detailed understanding of its safety and bioavailability, many pharmacokinetic studies and clinical trials of rhein have been done. Heo et al. used a physiologically based pharmacokinetic (PBPK) model of rhein to predict human pharmacokinetics. They observed that when it was taken orally by rats, rhein was rapidly absorbed and then was mainly subjected to conjugations such as glucuronidation and sulfation, indicating that phase II {UDP-glucuronosyltransferase (UGT) and sulfotransferase (SULT)} in hepatic systems were the predominant metabolic pathways responsible for the biotransformation of rhein. The fact that phase I (CYP 450) was weak in hepatic clearance reminds us to pay attention to the interaction of drugs especially that cleared only by CYP 450 when we put rhein into clinical application. Their experiment put forward the safety dose of rhein which can even be up to 600 mg for 1 day [32]. To directly observe the pharmacokinetics of rhein in human body, Zhu et al. selected eight healthy male volunteers to join in a prospective crossover study. All subjects received a single dose of rhubarb extract (50 mg*·*kg^−1^) on two separate occasions, once orally and once by a retention enema. Compared with retention enema administration, oral administration turned out to be more higher in the Cmax, AUC0-∞, and AUMC, which means retention enema administration of rhein is the better choice for patients [33].

In a randomized controlled clinical trial, the patients with type-2 diabetic nephropathy were treated by a rhein supplementation at the dose of oral 5 g per day for 2 months, 4 months, and 6 months. There was significant decrease of total cholesterol, triglycerides, and serum TGF-*β*1 expression after 4-month and 6-month treatment [34]. Retention enema of rhein with other conventional treatments has also been proved to bring down proteinuria, blood urea nitrogen (BUN), and serum creatinine (Scr), attenuating the renal function patients, which means that rhein can be used as an adjuvant safe therapy for these patients [35]. Patients with overt DN who take orally a lecithinized formulation of rhein turned better through decreasing microglobulin blood beta 2 (*β*2MG), serum creatinine (Scr), serum cystatin C (CysC), and urinary albumin excretion rate (UAER) [36].

## 7. Conclusion

A lot of scientific researches demonstrate that rhein has the ability to mediate multiple molecular targets, particularly oxidative stress and inflammation, which makes rhein a potential candidate for the therapy of some diseases, such as DN. In addition, rhein is characterized by mature extraction method, abundant resources, good tolerability, low price, and toxicity; thus, so many advantages can make it get more extensive application in clinical prevention and treatment in the future. Despite all of these big findings, more comprehensive and well randomized controlled human studies are still needed to explore.

## Figures and Tables

**Table 1:** The pathogenesis of DN.

Genetic predisposition	Renal hemodynamic changes [37]
ACE genetic polymorphism [38]	Vasoactive hormones
MTH-FR genetic polymorphism [38]	Systemic and intraglomerular pressure
AGT genetic polymorphism [39]	Activation of RAAS
Apolipoprotein E genetic polymorphism [40]	Oxidative stress [41]
	Nicotinamide adenine dinucleotide phosphate (NADPH)
Aldose reductase (ALR2) genetic polymorphism [42]	
	Reactive oxygen species (ROS)
Genetic locus 10p15.3, 7q21.3, 18q22.3, 14q23.1 [43]	Glucose-6-phosphate dehydrogenase (G6PDH)
	Inflammatory reaction [44]
Abnormal glucose metabolism [45]	C-reactive protein (CRP) [46]
Advanced glycation end products (AGEs) formation	Intercellular adhesion molecule (ICAM)
	Interleukin-1 (IL-1)
	Monocyte chemotactic protein-1 (MCP 1)
Hexosamine pathways increased	Tumor necrosis factor-*α* (TNF-*α*)
Polyol pathway flux increased	Cytokine
Protein kinase C (PKC) activation	Connective tissue growth factor (CTGF) [47]
Renal lipid accumulation	Insulin-like growth factor-I (IGF) [48]
Adenosine monophosphate activated protein kinase (AMPK) [49]	Transforming growth factor-*β* (TGF-*β*) [50]
Sterol regulatory element-binding protein (SREBP) [51]	Vascular endothelial growth factor (VEGF) [52]
